# Pure Nodal Small Lymphocytic Lymphoma: Clinical, Pathologic, and Outcome Features in a Single-Center Cohort

**DOI:** 10.3390/medicina62061200

**Published:** 2026-06-22

**Authors:** Andreea Georgiana Stoica, Mariana Așchie, Miruna Gherase-Cristian, Anca Florentina Mitroi, Georgeta Camelia Cozaru, Mădălina Boșoteanu, Cristina Cioti, Sorin Deacu, Irina Tica

**Affiliations:** 1 Faculty of Medicine, “Ovidius” University of Constanta, 900470 Constanta, Romania; georgiana-stoica@365.univ-ovidius.ro (A.G.S.);; 2 Center for Research and Development of the Morphological and Genetic Studies of Malignant Pathology-CEDMOG, “Ovidius” University of Constanta, 900591 Constanta, Romania; aschiemariana@yahoo.com (M.A.);; 3 Department of Hematology, “Sf. Apostol Andrei” Emergency County Hospital, 900591 Constanta, Romania; 4 Department of Clinical Pathology, “Sf. Apostol Andrei” Emergency County Hospital, 900591 Constanta, Romania; 5 Academy of Medical Sciences, 030167 Bucharest, Romania; 6 Department of Forensic Medicine, “Sf. Apostol Andrei” Emergency County Hospital, 900439 Constanta, Romania; 7 Department of Internal Medicine, “Sf. Apostol Andrei” Emergency County Hospital, 900470 Constanta, Romania

**Keywords:** small lymphocytic lymphoma, chronic lymphocytic leukemia, pure nodal SLL, TP53, time to first treatment, progression-only survival

## Abstract

*Background and Objectives*: Small lymphocytic lymphoma (SLL) represents the tissue-based manifestation of chronic lymphocytic leukemia (CLL). Despite their shared biological background, patients with SLL have been underrepresented in CLL-focused clinical trials, and data addressing the clinical behavior of pure nodal SLL remain scarce. The present study aimed to identify factors associated with time to first treatment (TTFT) and progression-only survival in patients with pure nodal SLL. *Materials and Methods*: In this prospective observational study, 46 patients with pure nodal SLL were included and followed for a median duration of approximately 5 years. Clinical, laboratory, histopathological, and TP53-related parameters were evaluated for their prognostic impact on TTFT and progression-only survival. *Results*: On univariable analysis, advanced-stage disease, hemoglobin < 10 g/dL, elevated serum β2M, elevated lactate dehydrogenase, del(17p), and aberrant p53 immunohistochemical expression were significantly associated with shorter TTFT and progression-only survival. *Conclusions*: Pure nodal SLL is a heterogeneous entity with a variable clinical course. Easily assessable clinical and biological parameters, including TP53 abnormalities, may help predict treatment requirement and disease progression, thereby contributing to better risk stratification and more individualized management. Kaplan–Meier analysis demonstrated significantly shorter time-to-first-treatment (TTFT) among patients with elevated β2M levels (≥3.5 mg/L), bulky lymphadenopathy (≥5 cm), and advanced-stage disease.

## 1. Introduction

According to the 2022 International Consensus Classification (ICC), chronic lymphocytic leukemia/small lymphocytic lymphoma (CLL/SLL) remains a single disease entity, with the distinction based primarily on the site of involvement: peripheral blood for CLL and lymph nodes/tissues for SLL [1]. CLL/SLL is a B-cell lymphoma comprising monomorphic small mature B cells that frequently co-express CD5 and CD23. A peripheral blood diagnosis of CLL requires a B-cell count of ≥5 × 10^9^/L, with the characteristic morphology and immunophenotype. A tissue-based diagnosis of SLL requires organ enlargement (e.g., lymphadenopathy > 15 mm) and its infiltration by the above neoplastic B cells. Although CLL and SLL represent the same disease, the latter term is used for cases with <5 × 10^9^/L circulating B cells in peripheral blood and nodal, splenic, or other extramedullary involvement [2,3].

SLL accounts for approximately 10–15% of cases within the CLL/SLL spectrum and represents around 5% of all non-Hodgkin lymphomas [4,5,6]. In clinical practice, many patients present with advanced-stage disease, commonly characterized by generalized lymphadenopathy, splenomegaly, hepatomegaly, and disease-related anemia.

In most clinical trials conducted in CLL, patients with SLL were either excluded or not clearly defined as a distinct subgroup. Paradoxically, patients with SLL were included in clinical trials evaluating chemotherapy in indolent non-Hodgkin lymphomas (iNHL) [7]. Although international guidelines recommend a similar management approach for both entities, available literature suggests that SLL is still frequently managed as an iNHL in real-world practice [8,9,10]. In this context, a better clinico-morphological characterization of pure nodal SLL and the identification of clinically relevant prognostic factors may improve risk stratification and support a more individualized therapeutic approach. Therefore, the present study aimed to identify factors influencing time to first treatment (TTFT) and progression-only survival in patients with pure nodal SLL and the heterogeneity of management in these patients in the real world.

## 2. Materials and Methods

### 2.1. Study Design

This is a single-center prospective observational study including 46 patients diagnosed with pure nodal B-cell small lymphocytic lymphoma in the Department of Pathology of the Emergency Clinical County Hospital of Constanta and subsequently followed and treated in the Hematology Department of the same hospital. We performed a prospective study over 6 years (2017–2022). Patient enrollment ended on 31 December 2022, and follow-up continued until 31 December 2024.

The main inclusion criterion was a diagnosis of pure nodal SLL established on the basis of histopathological and immunohistochemical examination (Biocare Medical, LLC; Irvine, CA, USA) together with the absence of peripheral lymphocytosis meeting the diagnostic threshold for CLL. To obtain a more homogeneous cohort, only cases with a characteristic nodal SLL phenotype were included. All cases showed proliferation centers and were positive for CD5, CD20, CD23, and LEF1, while negative for CD10 and cyclin D1. Cases with bone marrow involvement, as assessed by bone marrow biopsy, were excluded (“pure nodal” disease strictly). By using these criteria, we aimed to minimize diagnostic overlap with leukemic CLL presentations and with limited tissue involvement by CLL/SLL-like clones. Peripheral blood flow cytometry was routinely performed in all cases to exclude leukemic CLL presentations meeting diagnostic thresholds. Cases compatible with monoclonal B-cell lymphocytosis-like presentations were not included.

In all cases, p53 immunohistochemical expression was evaluated according to staining pattern and intensity. The p53 antibody from Zeta (Zeta Corporation, Sierra Madre, CA, USA), clone DO-07, isotype IgG2b, kappa, showing nuclear positivity, was used. Interpretation was performed using a pattern-based approach, as follows: (1) wild-type expression, defined as variable nuclear staining in 1–50% of tumor cells; (2) overexpression, defined as strong and diffuse nuclear staining in more than 50% of tumor cells; and (3) null pattern, defined as complete absence of staining in tumor cells in the presence of a positive internal control. The overexpression and null categories were considered abnormal p53 expression patterns.

Evaluation of del(17p13) was performed by fluorescence in situ hybridization (FISH) using a locus-specific probe for TP53 gene deletion (CytoCell for P53 gene deletions—TP53—LPS 037, Oxford Gene Technology IP Limited, Kidlington, UK) on formalin-fixed, paraffin-embedded tissue sections. Sample analysis was performed using a fluorescence microscope.

Our study was approved by the Ethics Committee of Emergency Clinical County Hospital of Constanta, and all the patients signed the informed consent at the time of hospitalization.

### 2.2. Patient Evaluation and Treatment Decision

All patients underwent clinical and paraclinical evaluation at diagnosis and were assigned disease stage and risk group accordingly. In patients requiring treatment initiation, the therapeutic approach was heterogeneous, and different treatment regimens were used. The median time from diagnosis to initiation of first-line therapy was 13 months (range, 1–84 months). Treatment approaches included chemoimmunotherapy-based regimens, monoclonal antibody-based therapy, and Bruton tyrosine kinase inhibitors, selected according to disease burden, clinical status, treatment availability, and physician judgment.

### 2.3. Statistical Analysis

Potential prognostic factors were assessed using Kaplan–Meier survival analysis and Cox proportional hazards regression in both univariable and multivariable analyses. A two-sided *p*-value < 0.05 was considered statistically significant for all tests. Cox proportional hazards assumptions were verified graphically and by testing Schoenfeld residuals. Variables with clinical relevance and/or significance in univariable analysis were included in multivariable models. Cases with missing data were excluded from the corresponding analyses. Cox proportional hazards assumptions were assessed using Schoenfeld residuals and graphical inspection. Variables with clinical relevance and/or significance in univariable analysis were entered into multivariable models. Missing data were handled by case-wise exclusion from the corresponding analyses.

Time to first treatment (TTFT) was defined as the interval from the date of diagnosis to the date of initiation of the first antineoplastic treatment. Patients who had not received treatment by the last follow-up visit were censored at the date of the last evaluation. Deaths occurring before treatment initiation were censored at the date of death, according to the study protocol.

In this study, progression-only survival was operationally defined as the interval from the date of diagnosis to the first documented disease progression. Patients without documented progression were censored at the date of the last evaluation confirming the absence of progression or at the date of the last visit/last contact. Patients who initiated antineoplastic treatment without documented progression were censored at the date of the last tumor assessment performed before treatment initiation. Because death was not included as an event in the present analysis, this endpoint reflects progression-only survival and should be interpreted cautiously, as it is not directly comparable to conventional progression-free survival definitions used in clinical trials.

Overall survival (OS) was defined as the interval from diagnosis to death from any cause. Patients who were alive at the end of follow-up or at the date of the last visit/last contact were censored at that time. Because of the relatively limited number of deaths observed during follow-up, OS analysis was considered descriptive and was not used as a primary endpoint. (For statistics, we used MedCalc Statistical Software Ltd., version 23.5.1 (Ostend, Belgium)).

## 3. Results

The median white blood cell count was 8940/μL (range, 3020–15,000/μL), while the median lymphocyte count was 3840/μL (range, 480–8070/μL). The baseline clinical and paraclinical characteristics of the study population are summarized in Table 1. The mean age of the patients was 65 years (range, 35–77 years). A total of 46 patients were included, of whom 16 were female and 30 were male.

According to the Ann Arbor classification, 18 patients (39%) were diagnosed with limited-stage disease and 28 patients (61%) with advanced-stage disease. B symptoms were present in 12 patients (26%) and were significantly associated with advanced-stage disease, as patients with stage III/IV disease were more likely to present with systemic symptoms (*p* = 0.002). Splenomegaly was identified in 25 patients (89.28%) in the advanced-stage subgroup, whereas only one patient with limited-stage disease had splenomegaly at diagnosis (*p* < 0.0001).

The distribution according to the International Prognostic Index (IPI) was as follows: low risk (IPI 0–1), 9 patients (19.6%); low-intermediate risk (IPI 2), 12 patients (26.1%); high-intermediate risk (IPI 3), 20 patients (43.5%); and high risk (IPI 4), 5 patients (10.9%). Most patients with advanced-stage disease were classified into higher-risk groups, and the distribution of IPI scores differed significantly according to disease stage (*p* < 0.0001).

Regarding nodal burden, 16 patients (34.78%) presented with lymphadenopathy < 5 cm, whereas 30 patients (65.21%) had lymph nodes measuring ≥ 5 cm at diagnosis. In stage III/IV disease, most patients presented with lymphadenopathy ≥ 5 cm (26 patients), and this association was statistically significant (*p* < 0.0001).

At diagnosis, 21 patients (46%) had a hemoglobin value < 10 g/dL. The minimum recorded hemoglobin value was 5 g/dL, the maximum was 16.2 g/dL, and the mean hemoglobin value was 10.74 g/dL. Patients with advanced-stage disease more frequently had hemoglobin values < 10 g/dL (60.7%) compared with those with limited-stage disease, among whom 83.3% had hemoglobin values > 10 g/dL. Therefore, low hemoglobin was associated with more advanced disease and an unfavorable clinical course (*p* = 0.0001). The relatively high frequency of anemia despite the exclusion of bone marrow involvement may reflect advanced systemic disease burden, inflammatory mechanisms, hypersplenism, or autoimmune phenomena in this selected cohort.

Serum β2M (β2M) levels < 3.5 mg/L, derived de novo by the Youden index, were more frequently observed in patients with limited-stage disease, whereas patients with advanced-stage disease more often had values > 3.5 mg/L, supporting the adverse prognostic value of elevated β2M (*p* < 0.0001). Similarly, serum LDH was normal in most patients with limited-stage disease (77.78%), whereas elevated LDH levels were more common in patients with advanced-stage disease (82.14%) (*p* < 0.0001). Both elevated serum β2M and elevated LDH were also associated with the presence of lymphadenopathy ≥ 5 cm (*p* = 0.002 and *p* = 0.0008, respectively). Elevated LDH levels may additionally reflect higher tumor burden or more biologically aggressive disease subsets in this tertiary referral cohort.

**Figure 1:**
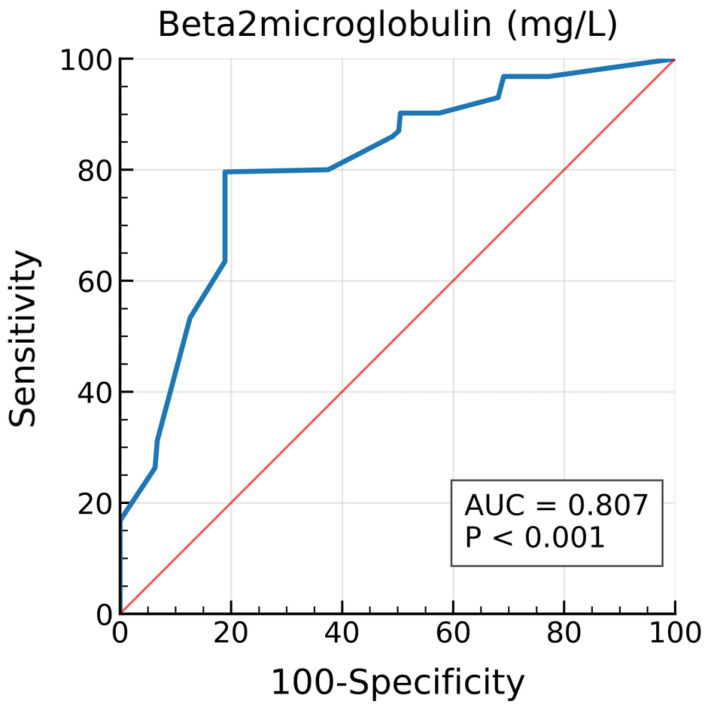
ROC curve for serum β2M in predicting advanced-stage disease. Blue is for Sensitivity and Red is for Specificity.

Receiver operating characteristic (ROC) curve analysis showed that serum β2M (see Figure 1) significantly discriminated between patients with Ann Arbor stage I/II and those with stage III/IV disease, with an area under the curve (AUC) of 0.876 (95% CI, 0.78–0.95; *p* < 0.001). The optimal cut-off value determined by the Youden index was 3.5 mg/L, corresponding to a sensitivity of 85% and a specificity of 80%.

According to the initial therapeutic approach, 12 patients (26.1%) were managed with a watch-and-wait strategy, whereas 34 patients (73.9%) required treatment initiation. Among treated patients, 15 (44.1%) achieved a complete response, while 19 (55.9%) were non-responders.

**Figure 2:**
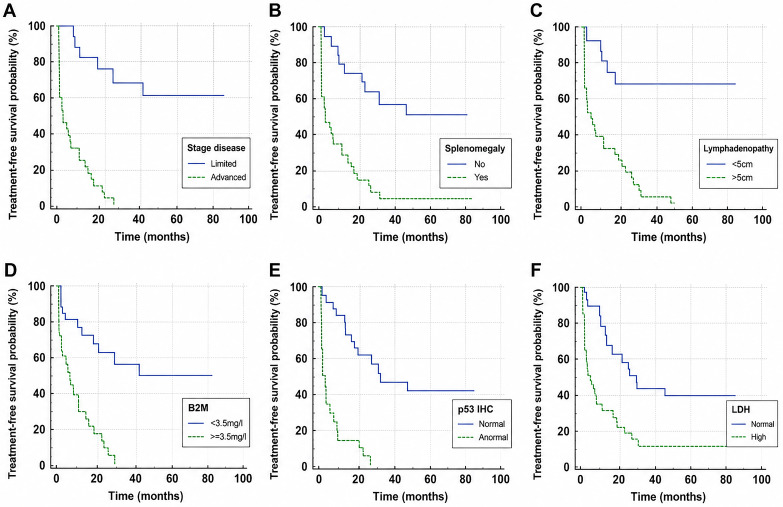
Univariate Kaplan–Meier analysis of time to first treatment (TTFT) according to selected clinical and paraclinical variables. Kaplan–Meier curves illustrate TTFT according to (**A**) disease stage, (**B**) splenomegaly, (**C**) lymph node size, (**D**) serum β2M level, (**E**) p53 immunohistochemical expression, and (**F**) LDH level.

In the univariable analysis (Figure 2), advanced-stage disease, splenomegaly, lymphadenopathy ≥5 cm, elevated serum LDH, elevated serum β2M, hemoglobin <10 g/dL, and TP53 abnormalities defined as del(17p13) and/or aberrant p53 immunohistochemical expression were associated with shorter TTFT.

In the multivariable model including clinical parameters, Ann Arbor stage remained an independent predictor of shorter TTFT (HR = 7.03; IC95%: 2.21–22.34; *p* = 0.0010). In the multivariable model including paraclinical parameters, β2M and hemoglobin remained independent predictors of shorter TTFT (HR = 2.55, IC95%: 1.06–6.15, *p* = 0.0365; and HR = 0.74, IC95%: 0.61–0.90, *p* = 0.0026, respectively).

With regard to TP53 abnormalities, both del(17p13) and aberrant p53 immunohistochemical expression (null pattern or overexpression) were significantly associated with a shorter TTFT (*p* < 0.0001). In the multivariable analysis, both variables remained independent predictors of earlier treatment initiation (*p* < 0.0001). Event rates and censoring distributions are summarized in Supplementary Table S1. 

Progression-only survival differed significantly according to the initial therapeutic approach. Kaplan–Meier analysis showed significant differences among the four treatment groups (*p* = 0.0062). The longest progression-only survival estimates were observed in the watch-and-wait group, in which no progression events were recorded during follow-up. However, this finding most likely reflects favorable baseline disease characteristics and lower disease burden in patients selected for observation rather than an effect of any therapeutic strategy. Similarly, differences observed among treated subgroups should not be interpreted as evidence of comparative treatment efficacy because treatment allocation was not randomized and was likely influenced by disease severity, patient characteristics, physician preference, and treatment availability.

Patients treated with chemotherapy alone experienced 8 progression events among 9 patients (88.9%), with a median progression-only survival of 27.13 months. Patients treated with chemoimmunotherapy experienced 12 progression events among 20 patients (60.0%), with a median progression-only survival of 36.17 months. In the BTKi group, 2 progression events were observed among 5 patients (40.0%), and the median progression-only survival was not reached. For the overall cohort, the median progression-only survival was 71.17 months. These descriptive findings should be interpreted with considerable caution. The study was observational, treatment allocation was not randomized, subgroup sizes were small and unbalanced, and treatment selection was likely influenced by baseline disease characteristics, disease burden, physician preference, and treatment availability. Consequently, the observed differences cannot be interpreted as evidence of comparative treatment efficacy and should be regarded as exploratory and hypothesis-generating only.

Concordance between p53 IHC and FISH del(17p) was significant, as all del(17p)-positive patients showed aberrant p53 IHC expression (sensitivity 100%; 95% CI: 78.2–100%). Thus, aberrant p53 IHC may be considered a practical screening tool for TP53 abnormalities; however, it does not replace comprehensive TP53 mutational testing. In addition, treatment response differed according to p53 IHC status, as patients with wild-type p53 expression had a complete response rate of 85.7%, compared with 15.0% among those with aberrant p53 expression. These findings suggest that abnormal p53 immunohistochemical expression may identify a subgroup of patients with less favorable responses to chemo(immuno)therapy. However, because comprehensive TP53 mutational analysis was not performed, the present data should not be interpreted as direct evidence of TP53-disrupted disease. Accordingly, p53 immunohistochemistry should be regarded as a screening or complementary biomarker rather than a surrogate for complete TP53 characterization.

## 4. Discussion

Pure nodal small lymphocytic lymphoma remains a relatively uncommon and insufficiently characterized presentation within the CLL/SLL spectrum. Although chronic lymphocytic leukemia and SLL are currently regarded as different clinical manifestations of the same biological entity, patients with SLL have historically been underrepresented in CLL-focused clinical trials, limiting the availability of subgroup-specific evidence [11].

A major strength of the present study is the strict case selection, aimed at defining a homogeneous cohort of true nodal SLL. In tissue-based presentations, distinguishing overt SLL from minimal or incidental tissue involvement by CLL-like clones may be challenging. By including only cases with characteristic histopathological features, including proliferation centers and a typical immunophenotype, and by excluding bone marrow involvement, we attempted to minimize diagnostic heterogeneity. However, this approach may also introduce selection bias toward a more narrowly defined disease subset, which should be considered when interpreting the results [12].

Our findings indicate that advanced Ann Arbor stage, splenomegaly, and lymphadenopathy ≥ 5 cm were associated with a shorter TTFT. These associations are biologically plausible, as they likely reflect increased tumor burden and a higher probability of clinically active disease. Similarly, the 2018 iwCLL guidelines define massive lymphadenopathy (≥10 cm) as a criterion of active disease requiring treatment. Contemporary data further support the clinical relevance of a ≥5 cm cutoff. In CLL14, baseline lymph node size ≥5 cm independently predicted shorter progression-free survival in patients treated with venetoclax–obinutuzumab, whereas in ibrutinib-treated cohorts, non-bulky disease was associated with a higher probability of complete response [13,14]. Bulky lymphadenopathy has also been classically linked to del(11q), which may partly account for the higher prevalence of large lymph nodes in advanced-stage disease [15].

Consistent with the 2022 International Consensus Classification for CLL/SLL, our findings support lymph node size as a surrogate measure of tumor burden with potential prognostic relevance. Although Ann Arbor staging and the International Prognostic Index were not originally developed for CLL/SLL, they appeared to capture disease extent reasonably well in this cohort. This observation aligns with the ICC concept that treatment decisions in CLL/SLL are driven primarily by evidence of active and clinically significant disease rather than by anatomical stage alone [2].

Among laboratory parameters, serum β2M and hemoglobin emerged as independent predictors of shorter TTFT. Elevated β2M likely reflects both tumor burden and adverse disease biology, while anemia at diagnosis may identify patients with more advanced or clinically significant disease. These findings are consistent with previous data in CLL and support their applicability in nodal SLL presentations. An additional finding deserving comment is the relatively high prevalence of anemia (46%) and del(17p) (30%) observed in our cohort compared with rates generally reported in unselected CLL/SLL populations. Several factors may have contributed to this observation. First, the study was conducted in a tertiary referral center, which may have resulted in preferential inclusion of patients with more advanced or clinically significant diseases. Second, the strict selection of patients with tissue-confirmed pure nodal SLL and exclusion of leukemic presentations may have enriched the cohort for individuals with a higher nodal tumor burden. Finally, the relatively small sample size may have amplified the impact of case-mix variation. Therefore, the frequencies of these adverse features should be interpreted cautiously and should not necessarily be considered representative of the broader SLL population.

TP53 abnormalities represent one of the most clinically relevant findings of this study. Both del(17p13) and aberrant p53 immunohistochemical expression were associated with earlier treatment requirements and retained significance in multivariable analysis. These results are consistent with the recognized adverse prognostic significance of TP53 abnormalities in CLL/SLL. Nevertheless, because TP53 mutational status was not systematically evaluated in the present cohort, our findings should be interpreted as reflecting associations with del(17p) and aberrant p53 immunohistochemical expression rather than with TP53 disruption as a whole. The observed concordance between del(17p) and aberrant p53 immunohistochemical expression in this cohort suggests that p53 immunohistochemistry may serve as a practical screening tool in routine settings. However, this observation should be interpreted with caution, given the limited sample size, and immunohistochemistry cannot replace comprehensive molecular testing for TP53 alterations [16].

The analysis of progression-free survival according to treatment strategy showed significant differences between subgroups; however, these findings should be considered exploratory. The more favorable outcome observed in the watch-and-wait group most likely reflects the selection of patients with less aggressive disease at baseline rather than a true therapeutic effect. In addition, the small size of treatment subgroups, particularly the BTKi cohort, and the heterogeneity of treatment approaches limit the ability to draw conclusions regarding comparative treatment efficacy. Therefore, no causal inferences regarding treatment benefit can be made based on these data. Any apparent differences between treatment groups should be regarded as hypothesis-generating only and require confirmation in larger prospective studies specifically designed for comparative treatment evaluation.

An important methodological aspect of this study is the definition of progression-only survival as a progression-based endpoint that does not include death as an event. While this approach allows a focused assessment of disease dynamics in this observational setting, it differs from standard definitions used in clinical trials and limits direct comparability with published data. This limitation should be taken into account when interpreting survival outcomes.

Overall, our results support the concept that pure nodal SLL is not uniformly indolent but rather exhibits substantial clinical heterogeneity. Readily available clinical and biological parameters, including tumor burden indicators and TP53 status, may help identify patients at higher risk of early treatment requirements and disease progression. These findings highlight the importance of an integrated clinicopathological approach to risk stratification in this rare presentation.

This study has several limitations, including its single-center design, the relatively small sample size, and treatment heterogeneity. In addition, TP53 assessment was limited to del(17p) and p53 immunohistochemistry, without comprehensive mutational profiling. Despite these limitations, the study provides real-world data in a rare and underreported subgroup and may serve as a basis for future multicenter investigations. The relatively high prevalence of adverse biological and clinical features, including anemia and del(17p), may also reflect referral and selection biases, potentially limiting the generalizability of the findings to less selected SLL populations.

Additional prognostic biomarkers currently used in CLL/SLL risk stratification, including IGHV mutational status, del(11q), trisomy 12, and del(13q), were not systematically available in this cohort and therefore could not be incorporated into the analysis. Consequently, the proposed prognostic assessment model should be interpreted cautiously and viewed as exploratory rather than definitive. Because of the limited number of death events recorded during follow-up, overall survival analysis remained descriptive and was not sufficiently powered for robust multivariable modeling.

## 5. Conclusions

Pure nodal small lymphocytic lymphoma represents a clinically heterogeneous entity rather than a uniformly indolent disease. In this cohort, advanced-stage disease, increased tumor burden, anemia, elevated serum β2M, and TP53 abnormalities were associated with a shorter time to first treatment and an unfavorable clinical course.

These findings support the integration of clinical, laboratory, and pathological parameters into the initial assessment of patients with nodal SLL, with particular emphasis on assessment of TP53-related abnormalities prior to treatment initiation. From a practical perspective, p53 immunohistochemistry may represent a useful screening tool; however, it should be considered complementary to, and not a substitute for, standardized molecular testing.

Importantly, the results of this study suggest that pure nodal SLL should not be managed solely within the framework of indolent lymphomas but rather approached within the broader biological and therapeutic context of CLL/SLL. Given the methodological limitations, including the small sample size and observational design, these findings require validation in larger, preferably multicenter studies.

## Figures and Tables

**Table 1:** Baseline clinical characteristics of patients with pure nodal SLL.

Patient Characteristic	n (%)
**Age**	
<60 years	11 (24)
≥60 years	35 (76)
**Sex**	
Female	16 (35)
Male	30 (65)
**B symptoms**	
Yes	12 (26)
No	34 (74)
**Splenomegaly**	
Yes	26 (57)
No	20 (43)
**Hepatomegaly**	
Yes	22 (48)
No	24 (52)
**Ann Arbor stage**	
Limited (I/II)	18 (39)
Advanced (III/IV)	28 (61)
**Lymphadenopathy**	
<5 cm	16 (35)
≥5 cm	30 (65)
**Performance status**	
0–1	29 (63)
≥2	17 (37)
**IPI**	
Low (0–1)	9 (20)
Low-intermediate (2)	12 (26)
High-intermediate (3)	20 (43)
High (4)	5 (11)
**Hemoglobin**	
≥10 g/dL	25 (54)
<10 g/dL	21 (46)
**LDH**	
Elevated	27 (59)
Normal	19 (41)
**βeta2-microglobulin**	
Elevated	23 (50)
Normal	23 (50)
**del(17p)**	
Present	14 (30)
Absent	32 (70)
**p53 (IHC)**	
Abnormal expression (overexpression/null pattern)	20 (44)
Normal expression	26 (56)
**Therapeutic approach**	
Watch and wait	12 (26)
CIT	29 (62)
BTKi	5 (11)

Abbreviations: β2M—beta2microglobulin; BTKi—Bruton tyrosine kinase inhibitor; CIT—chemoimmunotherapy; del(17p)—deletion of the short arm of chromosome 17; IHC—immunohistochemistry; IPI—International Prognostic Index; LDH—lactate dehydrogenase.

## Data Availability

The original contributions presented in this study are included in the article/Supplementary Material. Further inquiries can be directed to the corresponding authors.

## Supplementary Materials

The following supporting information can be downloaded at https://www.mdpi.com/article/10.3390/medicina62061200/s1; Table S1: Event and censoring distribution according to prognostic subgroup.

## Abbreviations

The following abbreviations are used in this manuscript:

AUC	Area under the curve
β2M	Beta-2 microglobulin
BTKi	Bruton tyrosine kinase inhibitor
CI	Confidence interval
CIT	Chemoimmunotherapy
CLL	Chronic lymphocytic leukemia
CLL/SLL	Chronic lymphocytic leukemia/small lymphocytic lymphoma
CR	Complete response
del(17p)	Deletion of the short arm of chromosome 17
del(17p13)	Deletion of chromosome 17p13
FISH	Fluorescence in situ hybridization
HR	Hazard ratio
IHC	Immunohistochemistry
iNHL	Indolent non-Hodgkin lymphoma
IPI	International Prognostic Index
iwCLL	International Workshop on Chronic Lymphocytic Leukemia
LDH	Lactate dehydrogenase
LEF1	Lymphoid enhancer-binding factor 1
mAb	Monoclonal antibody
OS	Overall survival
PFS	Progression-free survival
ROC	Receiver operating characteristic
SLL	Small lymphocytic lymphoma
TP53	Tumor protein p53
TTFT	Time to first treatment
WHO	World Health Organization
WW	Watch and wait

## References

[1] Campo E., Jaffe E.S., Cook J.R., Quintanilla-Martinez L., Swerdlow S.H., Anderson K.C., Brousset P., Cerroni L., de Leval L., Dirnhofer S. (2022). The International Consensus Classification of Mature Lymphoid Neoplasms: A report from the Clinical Advisory Committee. Blood.

[2] Naresh K.N., Ferry J.A., Rossi D., Geddie W.R., Wu C.J., Rawstron A.C., Eichhorst B., Akinola N.O., Yang S., Chiattone C., Schuh A., Coupland S.E., Sewell W.A. (2024). Chronic lymphocytic leukaemia/small lymphocytic lymphoma. WHO Classification of Haematolymphoid Tumours.

[3] Bahler D.W., Aguilera N.S., Chen C.C., Abbondanzo S.L., Swerdlow S.H. (2000). Histological and immunoglobulin VH gene analysis of interfollicular small lymphocytic lymphoma provides evidence for two types. Am. J. Pathol..

[4] Ben-Ezra J., Knowles D.M. (2001). Small lymphocytic lymphoma. Neoplastic Hematopathology.

[5] Tsimberidou A.M., Wen S., O’Brien S., McLaughlin P., Wierda W.G., Ferrajoli A., Faderl S., Manning J., Lerner S., Mai C.V. (2007). Assessment of chronic lymphocytic leukemia and small lymphocytic lymphoma by absolute lymphocyte counts in 2,126 patients: 20 years of experience at the University of Texas M.D. Anderson Cancer Center. J. Clin. Oncol..

[6] Martínez-Trillos A., Pinyol M., Delgado J., Aymerich M., Rozman M., Baumann T., González-Díaz M., Hernández J.M., Alcoceba M., Muntañola A. (2018). The mutational landscape of small lymphocytic lymphoma compared to non-early stage chronic lymphocytic leukemia. Leuk. Lymphoma.

[7] Rummel M.J., Niederle N., Maschmeyer G., Banat G.A., von Grünhagen U., Losem C., Kofahl-Krause D., Heil G., Welslau M., Balser C. (2013). Bendamustine plus rituximab versus CHOP plus rituximab as first-line treatment for patients with indolent and mantle-cell lymphomas: An open-label, multicentre, randomised, phase 3 non-inferiority trial. Lancet.

[8] Puckrin R., Owen C., Stewart D., Peters A. (2023). Real-world management of small lymphocytic lymphoma as chronic lymphocytic leukaemia versus an indolent non-Hodgkin lymphoma. Br. J. Haematol..

[9] Sachanas S., Pangalis G.A., Fink A.-M., Bahlo J., Fischer K., Levidou G., Kyrtsonis M.-C., Bartzi V., Vassilakopoulos T.P., Kalpadakis C. (2019). Small lymphocytic lymphoma: Analysis of two cohorts including patients in clinical trials of the German Chronic Lymphocytic Leukemia Study Group (GCLLSG) or in real-life outside of clinical trials. Anticancer Res..

[10] Olszewski A.J., Shafqat H., Ali S. (2015). Disparate survival outcomes after front-line chemoimmunotherapy in older patients with follicular, nodal marginal zone, and small lymphocytic lymphoma. Leuk. Lymphoma.

[11] Puckrin R., Owen C., Peters A. (2025). Underrepresentation of small lymphocytic lymphoma in clinical trials for chronic lymphocytic leukemia. Eur. J. Haematol..

[12] Gibson S.E., Swerdlow S.H., Ferry J.A., Surti U., Cin P.D., Harris N.L., Hasserjian R.P. (2011). Reassessment of small lymphocytic lymphoma in the era of monoclonal B-cell lymphocytosis. Haematologica.

[13] Pflug N., Bahlo J., Shanafelt T.D., Eichhorst B.F., Bergmann M.A., Elter T., Bauer K., Malchau G., Rabe K.G., Stilgenbauer S. (2014). Development of a comprehensive prognostic index for patients with chronic lymphocytic leukemia. Blood.

[14] O’Brien S.M., Jaglowski S., Byrd J.C., Bannerji R., Blum K.A., Fox C.P., Furman R.R., Hillmen P., Kipps T.J., Montillo M. (2018). Prognostic factors for complete response to ibrutinib in patients with chronic lymphocytic leukemia: A pooled analysis of two clinical trials. JAMA Oncol..

[15] Döhner H., Stilgenbauer S., Benner A., James M.R., Weilguni T., Bentz M., Fischer K., Hunstein W., Lichter P. (1997). 11q deletions identify a new subset of B-cell chronic lymphocytic leukemia characterized by extensive nodal involvement and inferior prognosis. Blood.

[16] Malcikova J., Pavlova S., Baliakas P., Chatzikonstantinou T., Tausch E., Catherwood M., Rossi D., Soussi T., Tichy B., Kater A.P. (2024). ERIC recommendations for TP53 mutation analysis in chronic lymphocytic leukemia—2024 update. Leukemia.

