# Supplementary material for: Pure Nodal Small Lymphocytic Lymphoma: Clinical, Pathologic, and Outcome Features in a Single-Center Cohort
Source: Medicina (Kaunas). 2026 Jun 22;62(6):1200. doi: 10.3390/medicina62061200 (PMC13304437; doi:10.3390/medicina62061200)
Supplement: Supplementary file 1 [file medicina-62-01200-s001.zip › medicina-4299022-supplementary.pdf]

Table S1. Event and censoring distribution according to prognostic subgroup.

Variable	Events (N)	Events (%)	Censored (N)	Total
β2M <3.5 mg/L	11	47.83%	12	23
β2M ≥3.5 mg/L	23	100%	0	23
Lymphadenopathy <5 cm	5	31.25%	11	16
Lymphadenopathy ≥5 cm	29	96.67%	1	30
Limited-stage disease	6	33.33%	12	18
Advanced-stage disease	28	100%	0	28
